# Lived experience of medical management in recurrent vulvovaginal candidiasis: a qualitative study of an uncertain journey

**DOI:** 10.1186/s12905-022-01973-x

**Published:** 2022-09-19

**Authors:** Moira Bradfield Strydom, Ramesh L. Walpola, Sara McMillan, Sohil Khan, Robert S. Ware, Evelin Tiralongo

**Affiliations:** 1 School of Pharmacy and Medical Sciences, Griffith University, Gold Coast, QLD Australia; 2 School of Population Health, Faculty of Medicine and Health, UNSW Sydney, Sydney, NSW Australia; 3 Menzies Health Institute Queensland, Griffith University, Gold Coast, QLD Australia

**Keywords:** Recurrent vulvovaginal candidiasis, Recurrent thrush, Women's health, Sexual health, Intimate relationships, Holistic healthcare

## Abstract

**Background:**

Recurrent vulvovaginal candidiasis (RVVC) is experienced by up to 10% of pre-menopausal women globally, yet there is limited research exploring the perspective of women living with this challenging condition.

**Methods:**

Semi-structured interviews with Australian women experiencing RVVC were conducted between April–July 2021. Interviews were transcribed verbatim, and qualitative interpretative phenomenological analysis (IPA) was conducted.

**Results:**

Ten RVVC patients were interviewed. IPA revealed an uncertain journey living with RVVC for all participants ranging from initial symptoms and difficulties in obtaining a diagnosis, the trial and error of symptom management, to the overall debilitating impact of living with a personal and intimate health condition. Four key themes were identified: Theme 1 outlined challenges and delays in diagnosis and clinically appropriate management. Theme 2 found that health care professional (HCP) knowledge limitations impacted RVVC management. Theme 3 illustrated the consequences of a lack of HCP support leading to self-referral and self-education. Theme 4 details the significant emotional and psycho-social repercussions of RVVC.

**Conclusions:**

This debilitating, life-long disease has a prolonged effect on women both physically and psychologically. Living with RVVC seems an uncertain journey that, to a large degree, women feel they must navigate alone. While resilience and self-empowerment were noted, better support through evidence-based treatment options, educated and evidence-informed HCPs and a sympathetic social support network is needed to decrease the disease burden. Future clinical management guidelines and patient support need to consider the findings of this study.

**Supplementary Information:**

The online version contains supplementary material available at 10.1186/s12905-022-01973-x.

## Background

Recurrent vulvovaginal candidiasis (RVVC) is a chronic subtype of vulvovaginal candidiasis (VVC) defined as at least four symptomatic episodes of VVC in the previous 12 months with at least one positive culture [1, 2]. Vulvovaginal pruritus, irritation, soreness, dyspareunia and vaginal discharge are considered to be cardinal symptoms, although are often variable in severity [3]. Idiopathic RVVC occurs in otherwise well individuals with no significant identifiable triggers such as antibiotic use or metabolic disorder [4]. Current estimates indicate that 75% of all women will develop VVC in their lifetime, with around 5–10% of women experiencing RVVC [5]. There has been no substantial reduction in lifetime annual prevalence rates of RVVC in the past 30 years despite advances in drug development and the introduction of long-term azole treatment. Projections suggest an upward trend in RVVC prevalence by 2030 [1].

As with other chronic women's health conditions (e.g. endometriosis), significant delays in diagnosing RVVC are common [6]. A diagnosis of RVVC often follows a substantial healthcare journey involving recurrent visits to a General Practitioner (GP) either from the onset of VVC symptoms or when symptoms recur following over-the-counter (OTC) treatment [7]. Management of RVVC is often sub-optimal, with no fully curative approaches in conventional medicine [8]. Prolonged courses of oral azole or vaginal therapy for 6 months or more, referred to as maintenance therapy, is the most effective management approach, which, after cessation, has an approximate 50% relapse rate [9]. A recent assessment of the prescribing guidelines for RVVC suggested a large variety in treatment approaches, leading to RVVC patients not being offered sufficiently prolonged maintenance therapy or even at all [10]. Referral pathways are also poorly defined, and referral is not always initiated [10]. Dissatisfaction with prescribed medical management, high relapse and patient doubts about medication safety and effectiveness have resulted in patient utilisation of complementary medicines (CM) [11, 12]. Health Care Practitioner (HCP) knowledge and care have been identified as important factors in the successful management of RVVC [7, 12, 13].

RVVC is a disorder with significant physical and psychological impact [14]. The repercussion of which extend to loss of confidence, self-esteem, intimacy and quality of life [1]. Qualitative studies have identified an undercurrent of shame and stigma [12, 13, 15, 16], as well as loss of productivity [14, 17], but the extent of the burden of the disease for patients living with RVVC is less studied.

This study explored the lived experiences of RVVC patients in Australia, including their perceptions of the journey to a diagnosis, medical management, and clinical care.

## Methods

### Design

An exploratory, qualitative study using Interpretative Phenomenological Analysis (IPA) was undertaken to identify the personal experience and lived realities of women with RVVC. Interpretive phenomenology is a well-established qualitative approach and is utilised here to provide a deeper understanding of the nature or meaning of RVVC patient experiences [18]. These experiences can be complex, emotionally laden, uncertain and reflective of an individual’s life experiences and responses to change. IPA allows thorough exploration of these subjective health experiences [18]. Griffith University human research ethical approval for the study was obtained (Ref No.:2021/144).

### Participants

Participants were recruited via email from a group of women over 18 years of age with a confirmed diagnosis of RVVC who had recently been screened for participation in a national RVVC clinical trial in Australia (ANZCTR 12620001084976). Women with idiopathic RVVC were recruited via purposive sampling to participate in semi-structured interviews. Idiopathic RVVC was defined as four or more episodes in a 12-month period, including evidence of at least one *Candida* positive vaginal culture and no other causative primary health conditions. Consent was obtained upon completing the pre-interview survey and verbally in the interviews. All participants were English speaking.

### Data collection

Participants completed a 10-min pre-interview survey using LimeSurvey [19] consisting of questions relating to demographic characteristics, concurrent health issues and sexual behaviour. One-on-one semi-structured interviews were conducted via Microsoft Teams within two weeks of the survey completion. An interview guide was developed to support the semi-structured interviews, containing questions regarding their first and recurrent episodes of RVVC; beliefs around the causes and triggers of RVVC; treatment options and management experiences; information access around self-help and CM utilisation; and the impact of RVVC on well-being (see Additional file 1: Interview Guide 1.0). The project team reviewed the guide and piloted it with consumers, colleagues and specialist clinicians working with RVVC.

Interviews occurred between April–July 2021. All interviews were undertaken by the same researcher (MBS) who had clinical and research experience with RVVC patients. Interviews were audio-visually recorded and transcribed via Microsoft Teams. Each participant was assigned a code, e.g. RVVCR1, RVVCR2, to ensure de-identification. Transcriptions were reviewed and quality checked manually by MBS to ensure engagement with the collected data and to maintain participant confidentiality. Notes were made during and after the interviews to assist understanding of data and analysis. All participants were sent de-identified copies of their transcript and provided the opportunity to review, change or add details as desired; one participant corrected a detail relating to dates and oral therapy.

### Data analysis

Transcripts were coded using NVivo (Version 1.5.1) by the interviewer (MBS) in consultation with the research team. IPA was performed to examine participant perspectives, highlighting differences and similarities and generating unanticipated insights [20]. Coded data and methods were independently reviewed by each member of the research team before being discussed on two occasions when discrepancies were resolved via consensus, coding was refined and re-organised, and the main emergent themes were defined.

Data were analysed simultaneously with data collection, the research team agreed that the 10th interview observed saturation consistent with other qualitative studies as no further themes were evident.

## Results

Twenty-eight RVVC patients identified through RVVC clinical trial screening were eligible; twelve patients completed the pre-interview survey. Two participants decided not to proceed with an interview because of personal reasons; their survey data was not included in the final analysis.

Pre-interview survey data revealed that participants were pre-menopausal and aged between 18 and 44 years. Nine participants were sexually active. A review of comorbidities revealed a range of conditions relevant to the pathophysiology of RVVC (Table 1). Eight participants reported two or more comorbidities in addition to their RVVC.

**Table 1:** Demographic and clinical characteristics of participants

RVVC participant characteristics	(n = 10) percentage	Frequency (percentage)
Age range of participants	18–24 years	1 (10%)
	25–34 years	4 (40%)
	35–44 years	5 (50%)
Sexually active	Yes	9 (90%)
	No	1 (10%)
Other health conditions	Irritable bowels syndrome	5 (50%)
	Anxiety	4 (40%)
	Depression	4 (40%)
	Hayfever	3 (30%)
	Recurrent urinary tract infection	3 (30%)
	Endometriosis	3 (30%)
	Adenomyosis	1 (10%)
	Polycystic ovary syndrome	1 (10%)
	Pelvic inflammatory disease	1 (10%)
	Small intestinal bacterial overgrowth	1 (10%)
	Inflammatory bowel disease	1 (10%)
	Psoriasis	1 (10%)
	Dermatitis	1 (10%)
	Autoimmune disorders—*(Hashimoto’s thyroiditis)*	1 (10%)

Analysis identified four key themes: (1) challenges with RVVC diagnosis and management, (2) limitations of HCP knowledge and management, (3) the impacts of lack of HCP support, and (4) emotional and psychosocial repercussions of living with RVVC.

## Theme 1: Challenges with RVVC diagnosis and management

All participants highlighted inconsistencies in HCP understanding of RVVC and diagnostic criterion, leading to difficulty and delay in obtaining the correct diagnosis and subsequent management. A common experience was the length of time it took to receive a diagnosis, which ranged from 9 to 48 months. This involved multiple appointments, repetitive investigations, and a perceived lack of awareness of the diagnostic criterion or seriousness of the condition by HCPs:

It took quite a while [for a diagnosis], probably nearly a year even. I still don't feel like anyone's taking it particularly seriously…I've had a million swabs and a million blood tests… It always comes back as the same thing, as Candida. (RVVCR15)

Participants reported that HCPs would perform repetitive swabs to confirm the presence of episodic *Candida* even though recurrent episodes were consistent with RVVC. Two participants felt their HCP did not follow structured diagnostic or testing guidance, which, together with frequent appointments with their HCP, contributed to a delay in their diagnosis:

So, she [doctor] said she would do her own swab and she said if it comes back positive for thrush, she'll send me to some sort of specialist for some sort of immunity thing… I just don't understand how that makes any sense, because I've had negative swabs this whole time… people I've spoken to about it have said that if you're treated with fluconazole, it's often hard to get a positive swab. (RVVC20)

Participants reported frustration, uncertainty, and disbelief about inadequate long-term medical management. This could be due to ineffective treatment strategies and limited HCP knowledge. Access to prescribed therapies for RVVC such as extended azole maintenance therapy varied for participants even with continuing care:

Nobody has actually offered me that [maintenance treatment]. Even the chemist has never mentioned it. Like, you know, when I go see the pharmacist for my creams, obviously I'm quite well known around the different shops when I go there. But nobody's said to me, hey, do you want to do this long term or anything like that. (RVVC21)

There was dissatisfaction with treatment options, including short-term and extended maintenance therapy. For example, some participants were frustrated with the lack of cure even when extended treatment was implemented:

It [fluconazole] did provide relief for a couple of days and then it wouldn't really do anything after that, like it would keep the symptoms sort of at bay. But they were still there. (RVVCR20)

Others expressed their reservations and dissatisfaction when faced with the realisation that treatment would have to be continued for years, possibly decades, but often saw no other option:

I think fluconazole is a pretty hardcore drug and telling someone to take that three to six months, this is one thing, but taking it once a week for the rest of your life just seems really, really full on. (RVVCR25)

I don't really like to take medications in general, and I know that those antifungals are a strong medication… I don't really want to, but if it gets rid of thrush, I'll do anything. (RVVCR16)

Being offered the same interventions repeatedly without permanent resolution resulted in treatment non-compliance for the following participant:

You know that I became non-compliant after a while, but I wouldn't go back to appointments 'cause I was just so sick of being bounced around and not having any results. (RVVC10)

Two participants expressed worry about using fluconazole for symptom control due to it being contraindicated in pregnancy, raising questions about ongoing treatment alternatives.

That's something that's tricky with this medication too, is we, you know, would potentially still like to have more children. But with this medication, you can't fall pregnant with it. I can't be on this medication all the time because if we were to fall pregnant. (RVVCR10)

## Theme 2: Limitations of HCP knowledge and management

A majority of participants viewed their GP as not specialised enough to support women experiencing RVVC with information or adequate treatment options:

I feel like a lot of the time I'm leading a lot of people [HCPs] into, where they should take me, particularly GPs. I feel like they know zero on this topic, you know, besides the standard thing. (RVVCR21)

Others felt that their HCPs approach to management was experimental and was associated with changing interventions frequently:

It feels like everybody's just trialling and erroring, and I've had to do a lot of my own research. (RVVCR20)

One participant sought specialist care from a gynaecologist, to feel let down at the lack of answers or advice provided. These interactions caused distress, and the lack of resolution for some participants added to the uncertainty of ever feeling well again. Some participants reported their HCP had acknowledged their knowledge limitations, suggesting that somebody more knowledgeable in RVVC would be better suited to continue their care:

…She [GP] said, you know, seeing as, we're not getting on top of it, you're probably going to have to go see a specialist, you have to work with somebody who kind of lives and breathes this... (RVVCR4)

However, referrals to HCP with specialist knowledge were not always successful:

Then after some time [I] went back to my GP because I wasn't feeling that I was getting heard from that gynaecologist. (RVVCR27)

Referral without benefit led some participants to seek help from other specialist practitioners. These included women’s health GPs, gynaecologists and vulval dermatologists, functional and integrative GP's, and CM naturopathic and nutritional practitioners. Yet, the issues with HCP knowledge were not restricted to conventional management pathways. One participant had some success with a Naturopath, only to find another had limited understanding of the condition:

She [naturopath] left the centre, I'm not sure where she went, and then, since then I've been with a new naturopath and she doesn't really know too much about it. (RVVCR20)

Despite high use of CM and some reported benefit, there was limited support for this from HCPs such as GPs and specialists. Participants experienced mixed reactions from their HCPs when questioned about alternative or adjunct treatment options:

My specialist, sort of laughs off any holistic…out that it's not proven and that her way is the only way that's actually going to work. (RVVCR10)

Sometimes HCPs dismissed interventions that have clinical evidence or stated that an intervention was dangerous when it was not:

I've tried probiotics myself. I just bought them from the chemist, but then one of the GPs that I saw, she was like don't take probiotics they're really bad and they make the thrush worse, I don't know the evidence, I just stopped taking them. (RVVCR16)

I asked my GP if I could use boric acid, and she said she doesn't prescribe it, she doesn't recommend it…after that, she spoke to her gynaecologist friend, who said, which was obviously the person I got referred to, who said, it's quite toxic not to use it. (RVVCR20)

Several participants tried modifying lifestyle factors like dietary and alcohol intake to reduce symptom flares. Those who discussed this approach and reported benefits to their HCP were dismissed. More than one participant reported being frustrated that no other options or additional information were offered by the HCPs as an explanation for this point of view:

And she [GP] was dismissive and said, oh, there's no evidence that diet is related, no clinical evidence of that, don’t worry about that. I could have taken that as her saying, eat whatever you want, drink whatever you want. It's not going to have any relationship to your vaginal health or the flora, which I just find remarkable because she didn't even explain anything further around her beliefs or the scientific research that she's come across for or against, you know, it was just dismissive. (RVVCR13)

For participants who had engaged with specialists and allied HCPs such as physiotherapists and psychiatrists, their recommendations appeared to primarily come from self-referral and self-education.

## Theme 3: Impact of lack of HCP support leading to self-education and self-referral

The burden of RVVC appeared to stem from not only the condition itself and the need to self-manage, but also from the interactions and general lack of support provided by HCPs. Participants reported helplessness, frustration and feeling let down by their HCP when discussing their overall management and journey with RVVC:

GPs, they don't seem to take it particularly seriously. It's more sort of like. Oh yeah. It's just Candida - do this, take this pill, you'll be fine. And then I'm back the next week and like no, it's still here. (RVVCR15)

Eight participants had a greater than three-year history of RVVC. During this time, these women had identified what works best for their condition and situation*.* Despite this, it was reported that HCPs would not include them in treatment decision making. This was particularly apparent if the HCP was an “available on the day” HCP:

They [GP's] don't listen, they don't understand the condition and they insist on choosing the treatment for me and would give me like a topical cream, which I say doesn't work for me… And it was really infuriating, again, because there was someone not listening to my clinical history or my experience and telling me what they know is best... (RVVCR13)

Incongruencies in RVVC knowledge and diverse management displayed by HCPs appeared to undermine the therapeutic relationship, thus adding to the psychological burden of the condition. One participant felt they were only taken seriously when their partner joined them at their appointment:

…I took my boyfriend with me because I just felt like I wasn't really being listened to in terms of my symptoms and stuff, and it's affected our relationship because its literally painful to have sex sometimes…it sort of seemed like that was the first time that my doctor took me seriously. (RVVCR20)

These types of interactions resulted in broken therapeutic relationships, leading to participants embarking on self-education and self-referral to manage disease burden.

Participants wanted to understand RVVC drivers and possible CM approaches more thoroughly. Many discussed links to hormones and menstrual cycles, allergic tendencies and immune systems, blood glucose levels, microbial colonies, pelvic floor tone, vaginal tissue health and pH within the vagina, thereby displaying a high level of understanding about the pathogenesis of RVVC. It was evident that the drive to educate themselves came from a lack of information from their HCP and the frustration of incomplete symptom resolution. Others felt that their HCPs approach was experimental and added to the burden of managing the condition as it led to frequently changing interventions and the need for self-education:

I was just being palmed off as if it was a once-off. You've just got thrush. You'll be fine. Take this treatment and then it'll go away. I'm sorry. It was like no one was listening, .... So, I started to do research on my own. (RVVCR27)

The desire to understand more led participants to seek and access online information from individuals they considered as knowledgeable in the area; this forum provided information on lifestyle, supplements and medicines that they felt was lacking from their HCP.

All participants had questioned why they experienced RVVC and what they were doing or had done in their lives that made them more at risk of experiencing symptom flares. This examination of personal attributes led to a range of lifestyle modifications, mostly after self-education, to provide symptom relief and prevent symptom flaring. Modifications to lifestyle factors were often not implemented under HCP guidance, online resources from others with similar experiences were frequently accessed:

I still feel like there's a lot of information lacking. So, for example, I would drive myself crazy trying to work out, you know, if I'm somehow reinfecting… every time it happened, I would hot wash my sheets, hot wash my towels, disinfect my lounge, and disinfect my chairs; it’s not like I sit on them without underwear…I would just go crazy with everything. (RVVCR15)

Participants reported requesting referrals to specialists such as gynaecologists or vulval dermatologists who may be able to recognise, differentiate and manage RVVC:

What if there is some other vulvar condition going on as well that would explain, like for people like me with Candida and negative swabs, even when there is copious amounts of classic discharge. Like how can that be a negative swab? I 'don't understand it. So, I took myself, I referred myself to a gynaecologist. (RVVC25)

So, when I went and saw the GP [general practitioner], I actually requested to go and see the gynaecologist because I'd never seen a gynaecologist before this point. (RVVCR21)

The burden of self-management and taking on their own care led to a financial burden as costs increased with trialling new practitioners and interventions. Cost of therapy was a consideration that prevented many from continuing care and long-term treatment with CM or prescribed medicine approaches:

The cost is what shocked me over the last couple of years, like I've spent between, like I'd say from about eight thousand [dollars] just on everything, trying to deal with it like and that's emergency appointments…your trying to get in before the weekend because, you know, it's flaring up and you feel like you just, you know, when you can't walk. It's just agony. (RVVCR4)

I want to express that it's insanely expensive; I must have spent well over a thousand dollars on this problem in the last 18 months. You know, for all the treatments. (RVVCR27)

The burden of unobtainable cure and associated financial repercussions were not the only negative aspect of treatment experienced by participants. Their recurrent symptoms had other far-reaching impacts as they changed their lifestyle and dealt with emotions associated with living with unresolvable symptom presentations.

## Theme 4: Emotional and psychosocial repercussions of living with RVVC

The array of negative emotions experienced by participants influenced their sense of self and identity and dictated interactions in social, professional, and intimate relationships. The majority of participants cited shame and awkwardness of this “personal issue”, and how it impacted so many facets of their life, describing this as overwhelming. All participants discussed their hesitancy to disclose the details of their condition to friends and family:

And I feel embarrassed talking about it. There is shame. I'm kind of letting go of that now, but it's always shameful thrush, like, I have that shame attached to it. I never felt comfortable talking to my girlfriends about it... (RVVCR25)

Participants reported interacting with others experiencing similar problems in online environments, often remaining anonymous. It allowed them to find out what had worked for others without the stigma and potential embarrassment of disclosure experienced in face-to-face interactions.

All participants in long-term relationships were thankful for spousal support and understanding. However, the experience was tempered by feelings of guilt related to not meeting partner sexual desire at the frequency they perceived appropriate or required to maintain a healthy sexual relationship. Most participants reported that despite the understanding displayed by partners, the restrictions placed on intimacy by RVVC caused distress, relationship strain and dissonance:

…I think my husband struggled, in the beginning, to understand that it [RVVC] was actually a thing; he’s like, you know, you should be over this by now… it's been four years... When I got it again, I'd get quite down. (RVVC27)

Yeah, but in terms of like sex and stuff like it's almost it's gotten to the point where my boyfriend is scared of my vagina…that's not really his fault. But, yeah, it definitely causes a strain. (RVVCR20)

Participating in sexual acts also contributed to the burden of symptom management by triggering the need to implement lifestyle routines post coitally. Additionally, pessimism was associated with the cessation of behaviours, such as wearing lingerie, perceived to be important for maintaining a healthy sexual relationship:

It definitely has a big impact on my sex life because I'm always thinking if we do some things, I'm thinking I'm going to get an infection. I thinking I'm going to have to go and have a shower or wash myself afterwards. Things like wearing, you know, some nice lingerie I never do that anymore because I know it's not good for me and it's just taken lots of things off the table and really created a mental barrier in my mind about doing sexual things because I'm always afraid that I'm going to get thrush again. It's just doesn't seem worth it. (RVVCR16)

A participant had specifically not sought out a new relationship because of the disclosure associated with their diagnosis and the limitations and physical compromises around intercourse:

If I met someone new, I would have to explain that situation to them and then I would have to go through the process I went through in the past relationship. (RVVCR27)

Trying one approach after the other, including implementing lifestyle change, caused not only a burden for themselves and their partners, but also impacted their whole family. Many participants expressed misgivings and annoyance associated with modifying so many aspects of their lifestyle and daily activities without guaranteed benefit. Participants went to extraordinary lengths of lifestyle changes, sometimes without guarantee of success, but were too scared to cease such activities for fear of symptoms worsening:

…Sometimes you get to a point, though, when you're too scared to go back just in case something is working. So, if I'm doing everything, you feel like 'you're doing a bit better’…And that just makes me too scared to change anything, I know there's probably tonnes of stuff that I'm doing that I don't need to be doing. I'm a little bit nervous, too, just in case 'it's the thing that's actually helping as well. (RVVCR4)

All participants were cognisant of the psychological and psychosocial burden that living with RVVC had on them. Despite this, none of the participants had discussed their vaginal health with a psychologist or similar professional, even if they were already accessing mental health support:

I think for me the psychological impact has been way bigger than I even realised. I think it all sort of started about yes 17/18 and even now at 34 [years of age], I'm still trying to figure it out and recover from it. (RVVCR10)

Nobody's ever said, how does this affect you mentally? Which, as I said, luckily, I'm not a depressive person because, you know, there's been times that I've been in tears over this, but it's tears of frustration. (RVVCR21)

But yeah, like, it's never occurred to me to get, I guess, counselling for that specifically, even though it's very distressing, I guess because it's so personal to, you know what I mean. You're the first person I've spoken to about it. Actually, I haven't even ever mentioned it to a friend. (RVVCR27)

Reluctance to discuss their condition even in the confines of professional confidentiality appeared to stem from the personal nature of RVVC.

## Discussion

This exploratory qualitative study provides insights to the life-long burden of living with RVVC. Major themes from present research highlights adverse psychological impacts stemming from delays to diagnosis, treatment programs, and the lack of understanding and referral from HCPs. Whilst patient’s struggles with RVVC are reported [7, 12, 21], the total extent of psychological, emotional and financial impacts on women living with RVVC remains understudied. This study also supports findings from studies conducted in similar cohort [1, 13, 17]. Surprisingly, our study findings suggest that the disease burden is primarily associated with apparent inadequacies in HCP knowledge of RVVC and the subsequent impacts on medical management.

There is a paucity of research exploring the diagnostic journey of RVVC patients timeframes from the onset of recurrent symptoms to receiving an RVVC diagnosis. Our findings highlight an extended timeframe to the diagnosis of up to three -years. Delay in diagnosis is commonly reported in women's reproductive health disorders like endometriosis and other vulval and genital related disorders such as Lichen sclerosis [6, 22]. Reasons for delay in diagnosis include non-specific and overlapping symptoms with other gynaecologic, urologic and gastrointestinal disorders, similar to RVVC [3, 6, 23] and isattributed with significant impacts on psychological wellbeing [6, 22]. Experiences shared by study participants identify a psychological impact from a lack of understanding by HCPs of RVVC which contributes to diagnostic delay and ineffective treatment. The diagnosis of RVVC requires an awareness of the difference between VVC and RVVC, a sound patient history to identify the frequency of symptomatic episodes coupled with an understanding of the limitations of culture-based microbial identification and previous response to azole therapy [3, 24]. Whilst evidence assessing HCP knowledge surrounding RVVC diagnosis is sparse, participants’ experiences would suggest that one or more of these factors impacted their diagnostic experience.

Interviews revealed that there was a wide variation to treatment approaches. Current evidence identifies maintenance therapy approaches of 6 months or more as efficacious for long-term symptom relief [10]. Maintenance therapy courses were often less than 6-months in duration as reported by study participants. The variations to maintenance therapy are consistent with a recent review of Australasian RVVC prescribing guidelines [10].

Participants perceived therapeutic management approaches for RVVC with mixed outcomes and feelings. While some were appreciative of the relief obtained from therapy others were concerned and confused, which led them to initiate CM approaches. Participants expressed safety concerns for over-reliance on oral azole as a long-term therapy and medications for RVVC in pregnancy and conception. The complexities in using pharmacotherapy options and their safety profile caused distress, as only topical azole therapy is considered safe in pregnancy [25, 26]. The risk of more severe symptoms in pregnancy due to estrogen levels and immune status [25, 27], and the inability to use oral azole therapy posed concerns as participants reported topical azole therapy was often an inadequate option.

A loss of confidence in HCPs was evident in the interviews, with many participants reporting either observed knowledge limitations or admittance from their HCP that they had reached their limit in being able to offer care. The dissatisfaction associated with the lack of knowledge of clinicians who treat RVVC as “just thrush” led to participants seeking repetitive OTC care and reliance on pharmacist advice. Both scenarios caused distress for individuals and often initiated ad-hoc referral to another practitioner or via the participants' request. Prior research suggests that positive and informed HCP experiences could strengthen the belief in the patient’s treatment journey [12]. Conducive communication with empathy and care was perceived as a respite from the challenging reality of the condition [7, 12] Majority of study participants felt their HCP was not as supportive as they were expected to be. Some participants who sought support from the same HCP still felt their presentation was downgraded to a minor complaint. Repercussion of such perception perpetuated diagnostic delays and decreased confidence in the HCP[12]. Participants’ who reported positive HCP interactions had regular care providers who had customised their management journey based on their clinical history. These positive interactions are reflective of HCP continuity of care strengthening the therapeutic relationship [12, 28]. Continuity is disrupted in a healthcare model where individuals lack a regular clinician, accessing next available HCPs [29]. Participants in our study discussed barriers to accessing effective treatments from “available on the day” GPs.

Our study also highlights lack of knowledge in holistic care approaches, including lifestyle, CM and allied health services. Eight participants had utilised CM nutritional and herbal supplemental therapy, with five participants accessing naturopathic care. Participants found these approaches to be supportive and, in some cases, preferred for their ability to control and manage recurrent symptom relapse as a component of person-centred care (PCC), where patient autonomy and care preference are essential [30]. Unfortunately, participants reported clinicians were not always supportive of CM therapies and, in some cases, dismissive of their potential role in RVVC management. A published survey of vulval health clinicians emphasised the important role of CM to complement conventional pharmacotherapies, with approximately 50% of their patients on probiotic therapy and 27% on dietary therapy [11]. These discrepancies may be influenced by the lack of vulval health and RVVC specialisation of the HCPs consulted by our participants.

The quality of life impacts of RVVC extend beyond experiences with HCP interactions, and diagnostic and therapeutic uncertainty. Diminished responses to physical and psychological well-being, in addition to effects on social relationships and overall loss of productivity, are attributed to poor quality of life with RVVC [14, 17]. Participants reported negative psychological impacts and uncertainty associated with ongoing lifestyle management and modification to reduce symptom severity and recurrence. Despite long perpetuated recommendations associated with personal hygiene, exercise, underwear fabric choice, lubricants and post-coital routines, there is little evidence to support many of these modifications [31–33]. Consistent with prior literature, these changes were introduced either without HCP knowledge or when HCP were aware they did not provide input or support [12]. For many of our participants, symptoms persisted despite changing multiple perceived influences. When minor improvements occurred, identifying which factor was beneficial was difficult. The inability to find one causative influence created a reluctance to modify anything in case symptoms worsened. Changes implemented included lifestyle factors with more conclusive evidence, such as diet and alcohol intake [31]. Whilst these changes appeared beneficial for symptom control, they had significant negative impacts socially and within the family unit. Consistent with earlier research more than one participant discussed missing out on social enjoyment or feeling burdensome to the family because of their self-imposed restrictions on diet and alcohol [12].

Clinically, RVVC psychological impacts are often overlooked, with symptoms perceived as insignificant and non-life impacting [6, 17, 34]. The need for psychological support in RVVC has been previously reported [34], although psychological support referral pathways do not feature in clinical practice guidelines [35]. These referral services could be utilised as an adjunct to pharmacotherapy while the patients navigate the uncertainty associated with unobtainable cure. The actual psychological burden of RVVC appears to be multifactorial; living with an incurable condition, disappointing and uncertain interactions with multiple HCPs, ongoing lifestyle modification and negotiation, and impacts related to intimate relationships and decreased self-esteem from self-imposed shame and stigma. Psychological stress is also considered as a risk factor RVVC, with sexual health, anxiety and depression impacting symptom recurrence [36, 37]. In our study, four participants reported having both anxiety and depression; of these two participants were accessing psychological support services, however, none were doing so to deal with the impacts of RVVC, nor had it been suggested to them by their HCP as a possible allied health therapy for RVVC management. Those accessing psychological support therapies cited shame and embarrassment, preventing them from discussing RVVC in their consultations.

Notwithstanding the significant impacts on intimacy and sexual health reported by participants and in qualitative studies exploring recurrent vaginal health issues [7, 38], no participants had accessed or been advised to access psychosexual or counselling support specifically for support in their intimate relationships. This lack of psychological support is congruent with participants noting that they had not discussed the condition's sexual health impacts with their HCP. There is an urgent need for allied health referral services in RVVC. Mental health care should be considered in RVVC management guidelines as a component of patient-centred care approach. The lack of allied health support service referrals suggests that the current treatment modality of RVVC is not aligned with the potential psychological implications of this condition on sexual health and relationships.

Despite what seemed like a disempowering and painful experience, participants displayed remarkable strengths. The empowerment comes from self-advocacy for referral and testing, from exploring difficult conversations with loved ones and family, and from pushing through, despite chronic relapsing symptoms to achieve life milestones related to family growth, career goals, and personal self-exploration. Participants also showed a significant understanding and empathy to the shortfalls apparent in the management of RVVC. HCPs who had provided them support despite cure remaining elusive were reported on favourably, highlighting the role of positive therapeutic relationships [28]. Collectively, participants were favourable towards a more integrative and patient-centred care approach that addresses the existing management gaps and causes of uncertainty in RVVC.

## Future directions

Holistic and integrative care is necessary as RVVC has a complex pathophysiology and clinical manifestation. As such identifying allied health and CM support services that can follow or complement conventional pharmacotherapy options is vital to improve clinical outcomes and offer additional support for relapsing patients. Educating HCPs on specific aspects of RVVC to support timely diagnosis and adequate management is crucial. Safety of pharmacotherapy and its place in specific conditions and situations such as pregnancy needs a clearer understanding. This also includes reviewing and updating clinical management guidelines to include referral pathways for diagnosis, management, and psychological support. The potential role of CMs and lifestyle modification as a component of patient-centred care need to be further explored. Patient-specific holistic care resources in an easy-to-understand format would strengthen confidence in clinical care, minimise patient concern and enhance wellbeing.

## Strengths and limitations

This study facilitated an open sharing of lived experiences for participants through a semi-structured interview format. Our study participants were driven by a need to find answers and better solutions for their chronic condition and may not be truly representative of all RVVC patient experiences. These participants may represent a subset of people for whom delay in diagnosis and problematic management is common, serving as motivation to seek solutions outside of standard medical care. This research was conducted from the patient perspective. To balance the perceived criticisms of HCPs and knowledge limitations assessing the experience and knowledge base of HCPs around RVVC management is required.

## Conclusion

Living with RVVC seems an uncertain, challenging, and lonely journey for most women. This research tells a compelling story of frustration, disappointment, dismay, and hopelessness compounded by delays, inadequacies, and inconsistencies associated with the long-term management of RVVC and the breakdown of the patient–practitioner relationship. While resilience and self-empowerment were noted, better support through evidence-based treatment options, educated and evidence-informed HCPs and an empathetic psycho-social support network is essential to decrease disease burden and improve health outcomes. The current and future RVVC management plan should consider: awareness by HCPs of RVVC as a chronic condition, its timely diagnosis and need for long-term management; effective pharmacotherapy options and support in utilising evidence-based lifestyle changes and timely referral to mental health and well-being support services.

## Supplementary Information

**Additional file 1.** Interview Guide 1.0. The interview guide was developed to support the semi-structured interviews, containing questions regarding their first and recurrent episodes of RVVC; beliefs around the causes and triggers of RVVC; treatment options and management experiences; information access around self-help and CM utilisation; and the impact of RVVC on well-being.

## Data Availability

The datasets generated and/or analysed during the current study are not publicly available due the personal nature of the data collected but are available from the corresponding author on reasonable request.

## Abbreviations

CM: Complementary medicine
GP: General practitioner
HCP: Health care professional
IPA: Interpretative phenomenological analysis
OTC: Over the counter
RVVC: Recurrent vulvovaginal candidiasis
VVC: Vulvovaginal candidiasis
