# Supplementary material for: Lived experience of medical management in recurrent vulvovaginal candidiasis: a qualitative study of an uncertain journey
Source: BMC Womens Health. 2022 Sep 19;22:384. doi: 10.1186/s12905-022-01973-x (PMC9487134; doi:10.1186/s12905-022-01973-x)
Supplement: Supplementary file 1 — Additional file 1. Interview Guide 1.0. The interview guide was developed to support the semi-structured interviews, containing questions regarding their first and recurrent episodes of RVVC; beliefs around the causes and triggers of RVVC; treatment options and management experiences; information access around self-help and CM utilisation; and the impact of RVVC on well-being. [file 12905_2022_1973_MOESM1_ESM.docx]

| **Additional File 1.0 Interview Guide** |
| --- |
| **ONSET**  What was happening in your life at the time you started to experience thrush/ (a few months before)?  People with recurrent thrush often have periods when they feel extreme symptoms, sometimes called flares. Do you often experience flare-ups? What is it like for you to experience flare-ups? Do you know what causes these flares?  Can you discuss other aspects of your health/life that you think impacts on or affects your RVVC? |
| **DIAGNOSIS**  Can you tell me about your experience leading up to and getting a diagnosis?  That sounds like it was (quite a journey/difficult) how does that make you feel?  How long did it take for you to get a diagnosis?  On reflection what did you think about that time frame?  Which health professionals were involved in obtaining your diagnosis of RVVC?  Was your doctor a specialist or a general practitioner? If you have seen a specialist about this condition, what sort of specialist?  You mention accessing treatment over the counter in a pharmacy, can you tell me whether you were recommended to see your GP for you recurrent thrush?  Did you see other health professionals for this issue in the lead up to your diagnosis? |
| **MANAGEMENT:**  **Medicinal product**  Can you discuss how you have managed RVVC using medicines? (This could include either prescription or pharmacy medicines, or complementary medicines such as for example probiotics, fish oil etc., or both)  **Specific prompts- Conventional Medicines/maintenance therapy/ complementary medicines as indicated by the answer to above**  **Conventional Medicines**  If you 'haven't tried conventional medicines for RVVC can you tell me why?  How do you feel about using conventional medications to manage RVVC?  Can you tell me what made you decide to use conventional medicine in the management of your RVVC?  With respect to medicine use, how involved were you in the decision-making process?  What are your thoughts on the amount of medicines information you were given by your doctor/prescriber/pharmacist?  Do you ever not treat the symptoms? If yes, are you able to explain why?  **Maintenance therapy- (for those who have confirmed they have used conventional medicine management).**  One of the approaches to recurrent thrush is called maintenance therapy consisting of longer courses of medicines for symptom relief. If you have used/using or been offered maintenance therapy for the management of RVVC how did you find this approach?  What was the length of time you tried this therapy for?  If you are no longer using this therapy why did you stop?  When you stopped the therapy how long until your symptoms returned if at all?  Can you tell me why you 'haven't tried this approach?  What are your thoughts on the impact of maintenance therapy on your health and well-being?  **Complementary medicines**  What sort complementary medicines have you used in the management of your RVVC and how effective did you find them?  Why have you utilised complementary or alternative medicines?  Where do you go to find information about these medicines?  What type of health professional, if any, has supported you with utilising complementary medicine?  What are your thoughts on the amount of medicines information you were given by these/this health professional/s?  If you 'haven't tried complementary medicines for RVVC can you tell me why?  Where do you purchase your complementary medicines from? |
| **ADDITIONAL THERAPIES AND SUPPORT SERVICES**  What other therapies (i.e meditation, massage etc) or support services have you used in the management of your RVVC and why?  What benefit, if any, do you think these services and support have provided you?  Is there a reason you 'haven't accessed or utilised any of these services?  Have you spoken to anyone about the psychological impacts of RVVC?  Who have you spoken to and what benefit if any did you find?  Why do you think you 'haven't talked to anyone about the impact of RVVC on your well-being?  Do you belong to any self-help / support groups in your community for RVVC? if yes - benefits? if not, reason? |
| **LIFESTYLE MODIFICATION**  What (if any) lifestyle changes have you made to manage your condition and what impact do you think those changes have made?  What influenced you to decide to make those changes?  How did changing (a, b,c,d,e) impact RVVC?   1. Diet 2. Clothing 3. Hygiene 4. Bathing practices 5. Sexual interactions |
